# Nursing and Municipal Economy

**Published:** 1919-11-22

**Authors:** 


### THE MATRONS' AND SISTERS' DEPARTMENT.

### NURSING AND MUNICIPAL ECONOMY.

It is a little unfortunate that a campaign for reducing the rates should be in progress exactly at the moment when important nursing reforms are crying aloud for the expenditure of large sums of money. Everyone knows that the care of the indoor sick poor has only hitherto been carried out effectually and at a low cost by the devotion of the nursing staff, from the matron downwards. Cut down to the lowest dimensions by guardians, with one eye always intent upon the rate-payers, the nursing staff has struggled on in Poor-law establishments all over the country on a scale of salaries pitifully inadequate. They have toiled early and late; they have foregone, not merely pleasure, but the most ordinary relief from monotony; they have been poorly housed, badly fed, and in some places very inadequately taught. But they have kept their institutions going, staved off disaster in many a time of extreme pressure, and shown individually and collectively a wealth of unselfish pity and devotion to their patients, only half guessed at by their managers, and too often entirely unknown to the public. All through the war they were being told "All will be quite different when the war is over." And now the day of reorganisation is at hand, and in every direction the managers of municipal hospitals are being urged to hold their hands lest they increase the rates. Is this quite fair to the nurses?

The public is beginning to be indignant about the "sweated" nurse. This is all to the good. But the public must be informed that to reduce nurses' hours, it is necessary to increase the number of nurses employed, and to do this means one of three alternatives. Either a fresh accommodation must be built for the additional women, or houses adjoining the institution must be rented for them, or part of the building now occupied by patients must be taken over for the staff, and the work of the institution must be thereby curtailed. There is no other way in which the eight-hour day, the one day's rest in seven, so pressingly demanded in the nurses' interests, can be secured.

The problem is a very urgent one and whatever plan be adopted, it spells extra expense. In the densely populated parts of London and provincial towns the initial difficulty of obtaining a site for building seems almost insuperable. At the Kensington Infirmary, for instance, where the housing difficulty is acute, there is not an inch of space in the grounds for the purpose of enlarging the Nurses' Home, nor is there any ground to be had adjacent. There is a limit to the extent to which fresh storeys can be added to existing buildings and most necessary reforms are being shelved everywhere for lack of a way out of this impasse. But brains and determination will find a way. Building, moreover, is not only infinitely more costly in itself, but the requirements of the nurses' home now exceed all that could have been conceived even twenty years ago. From fifty to a hundred thousand pounds is an ordinary estimate for a good-sized home, and even then there must always be space left for further enlargements. The plans of such homes, it must be remembered, aim at a severe simplicity and afford no more than the minimum of convenience and comfort for several hundred women living under the conditions proper to a modern establishment for the sick, with students in training. It is very doubtful if there be any real economy in hiring houses outside for the nursing staff. It is highly inconvenient from the administrative point of view, and, in many localities, it is impossible. It is desirable to do this, however, rather than delay reforms.

There remains the plan of adapting part of the patients' quarters to the uses of the staff, and reducing the amount of work which is undertaken. That may prove the most costly of all, as the results will show. For the institution exists primarily for the patients and nearly all infirmaries in crowded neighbourhoods are pressed for beds. Should a wing be put to other uses, there would soon arise the necessity for building another institution to meet the needs of the sick.

Under these circumstances the Health Ministry should take the public into counsel. The situation must be faced without delay. Unless the public is content to go on sweating the nurses by underpaying and overworking them, large sums of money must be found for enlarging existing Nurses' Homes or erecting new ones. Hundreds of Poor-law infirmaries all over the country are understaffed and are so badly constructed that the nurses suffer from imperfect sanitary conditions and dark, unwholesome, crowded quarters. This state of things cannot be altered without money, and there is no question but that the money must be supplied. The rate-payers have no right to press for economy in this direction until they have a clear conscience towards the nurses.

